# Evaluation and validation of HPV real-time PCR assay for the detection of HPV DNA in oral cytobrush and FFPE samples

**DOI:** 10.1038/s41598-018-29790-z

**Published:** 2018-07-27

**Authors:** Alexandre Harlé, Julie Guillet, Jacques Thomas, Xavier Sastre-Garau, Marie Rouyer, Carole Ramacci, Pauline Gilson, Cindy Dubois, Gilles Dolivet, Agnès Leroux, Julia Salleron, Jean-Louis Merlin

**Affiliations:** 1 Université de Lorraine, Nancy, France; 2 CNRS, UMR, 7039 CRAN Nancy, France; 3 Service de Biopathologie, Institut de Cancérologie de Lorraine, Vandœuvre-les-Nancy, France; 4 Unité de chirurgie cervico-faciale et odontologie, Institut de Cancérologie de Lorraine, Vandœuvre-les-Nancy, France; 5 Cellule data management et Biostatistique, Institut de Cancérologie de Lorraine, Vandœuvre-les-Nancy, France

## Abstract

Specific HPV genotypes have been recognized as risk factors inducing head and neck cancers (HNC). The aim of this study was to validate a real-time PCR assay to detect accurately High Risk HPV DNA in Formalin Fixed Paraffin Embedded (FFPE) and oral cytobrush samples and compare the results with conventional PCR. Repeatability, reproducibility and limit of detection of Cobas assay were estimated for oral cytobrush and FFPE samples of patients with HNC. 53 samples of patients with a HNC were then used for assay comparison with conventional PCR. Finally, 26 samples of patients with anogenital neoplasia cancer were analyzed as control and assays comparison. Among the 53 samples of patients with HNC, 12 (26.7%) were HPV positive, 33 (73.3%) were HPV negative and 8 (15.1%) were non contributive with the Cobas assay. Among the 26 samples of patients with anogenital neoplasia, 15 (57.7%) were HPV positive and 11 were HPV negative (42.3%). One sample was found with an HPV 16 and HPV 18 co-infection. Only 3 samples were found with discrepant results. Cobas assay was found suitable for routine HPV detection with a very good repeatability and reproducibility for all HPV genotypes (CV < 0.6% and <0.4% respectively). Sensitivity and specificity for Cobas assay were 91.7% [61.5%;99.8%] and 96.9% [83.8%;99.9%] respectively. Ten nanograms of DNA were sufficient for the detection of HPV 16, HPV 18 and HPV in FFPE and oral cytobrush samples. Cobas assay was found comparable to conventional PCR and can detect accurately and rapidly HPV DNA in FFPE and oral cytobrush samples for the management of HNC and other types of HPV-associated neoplasia.

## Introduction

Specific types of human papillomavirus (HPV) are recognized as major oncogenic factors for the development of a large subset of head and neck squamous cell carcinomas. More than 245 HPV types have been described as pathogens in human and at least 40 of them have a tropism for anogenital mucosa^1^. HPV are classified into high-risk (HR) and low risk (LR) according to their oncogenic potential, as shown in Suppl Table 1. HPV-16 is the most frequently identified genotype in head and neck cancers and has been recognized as human carcinogen in 1995 by the International Agency for Research on Cancer (IARC)^2^.

Over the last decade, the incidence of head and neck cancer has increased, despite the decreasing risk factors like tobacco smoking and alcohol consumption^3^. In parallel, HPV has been recognized as a potent risk factor inducing head and neck cancers, especially oropharyngeal squamous cell carcinomas in young male nonsmokers^4^. An international meta-analysis shows that HPV 16 is found in 87% of oropharyngeal cancers and in 68% of cancers of the oral cavity^5^.

Several studies indicate that oral and genital HPV infection are not independent: same subtypes are found in oral and genital tract in a same women or in sexual partners^6^. Cervical cytobrush sampling is the gold standard for the detection of dysplasia and cancers of the cervix. Unfortunately, there is no clinical routine used to detect oral or oropharyngeal dysplasia before their progression. Only a thorough clinical examination allows identification of suspicious lesions and biopsy is the only way to confirm diagnosis and search HPV infection. The most widely used method for the cervix is a liquid cytobrush sample: liquid-based cytology method using residual cell suspensions. This sample allows the cytology assessment of the sample and the research for an HPV infection. Nevertheless, this assay has not been designed or validated to assess oral cytobrush or Formalin Fixed Paraffin Embedded (FFPE) samples.

HPV status represents an independent prognostic factor in head and neck carcinoma, most probably related to the fact that HPV positive tumors are more sensitive to radiotherapy than HPV negative tumors^7^. FFPE tumor biopsies can be used for HPV detection or genotyping, but DNA can be altered or fragmented during the pre-analytical process and this may cause assays failure^8^. Few assays have been described for the detection and genotyping of HPV in FFPE^9^. The most common used and current validated assay is using PCR followed by a gel migration (conventional PCR)^10^ but requires several manual steps and more time than a real-time PCR.

The aim of this study was to validate a real-time PCR assay to detect quickly and accurately HR HPV in FFPE head and neck tumor samples and oropharyngeal cytobrush samples. We validated this assay according to ISO 15189 directives and French accreditation comity (COFRAC) recommendations. In this study, we adapted the CE-IVD marked Cobas HPV assay, which is validated for the detection and genotyping of HPV in cervix cytobrush samples, for the detection and genotyping of HPV in FFPE tumors and oral cytobrush samples. We finally analyzed with both conventional PCR and Cobas real-time PCR assays 53 FFPE and oral cytobrush samples of patients with head and neck cancer in double blind conditions and compared the results. Twenty-six consecutive samples of patients with anogenital neoplasia were also analyzed with both assays and used for confirmation.

## Results

### HPV genotypes

Samples with a possible DNA amplification were set as “interpretable” and samples with a too low DNA quality and no amplification as “non-interpretable”. Among the 53 head and neck assessed samples 45 had interpretable results with Cobas assay. HPV DNA was found in 12 samples (26.7%), 11 HPV16 (91.7%) and one HR HPV (8.3%). DNA was amplified but no HPV DNA was found for 33 samples (73.3%). Among the 53 assessed samples 44 had interpretable results with the conventional PCR and DNA amplification was not possible for 8 samples with Cobas assay and 9 with conventional PCR. HPV DNA was found in 12 samples (27.3%), 11 HPV16 (91.7%) and one HPV 6/11 (8.3%). No HPV DNA was found for 32 samples (72.7%).

Among the 26 anogenital neoplasia samples, HPV DNA was found in 15 samples (57.6%) and no HPV DNA in 11 samples (42.3%) with both assays. Cobas assay detected HPV16 DNA in 9 samples (60.0%), HPV 18 DNA in 5 samples (33.3%) and HR HPV DNA in 2 samples (13.3%). Conventional PCR detected HPV16 DNA in 8 samples (53.3%), HPV 18 DNA in 4 samples (26.7%) and HR HPV DNA in 5 samples (33.3%). Sample #3 was found with HPV 16 and HPV 18 with both assays (Suppl Table 2-b).

### Real-time PCR assay validation

Repeatability calculated CV for cytobrush samples was 0.48%, 0.32% and 0.59% for High Risk, HPV 16 and HPV 18 respectively. Repeatability calculated CV for FFPE samples was 0.25%, 0.60% and 0.31% for High Risk, HPV 16 and HPV 18 respectively (Table 1).

**Table 1:** Repeatability and reproducibility of Cobas HPV assay for the detection of HPV from DNA extracted from oral cytobrush and FFPE tissues.

	Repeatability						Reproducibility					
	Oral cytobrush			FFPE tissue			Oral cytobrush			FFPE tissue		
	High risk	HPV 16	HPV 18	High risk	HPV 16	HPV 18	High risk	HPV 16	HPV 18	High risk	HPV 16	HPV 18
mean Ct	25.26	22.68	23.98	26.98	20.19	30.85	23.60	20.89	21.70	26.98	20.26	30.99
standard deviation	0.12	0.07	0.14	0.07	0.12	0.10	0.09	0.19	0.10	0.10	0.08	0.04
CV	0.48%	0.32%	0.59%	0.25%	0.60%	0.31%	0.37%	0.90%	0.48%	0.37%	0.39%	0.12%

Reproducibility calculated CV for cytobrush samples was 0.37%, 0.90% and 0.48% for High Risk, HPV 16 and HPV 18 respectively. Reproducibility calculated CV for FFPE samples were 0.37%, 0.39% and 0.12% for High Risk, HPV 16 and HPV 18 respectively (Table 1).

**Figure 1:**
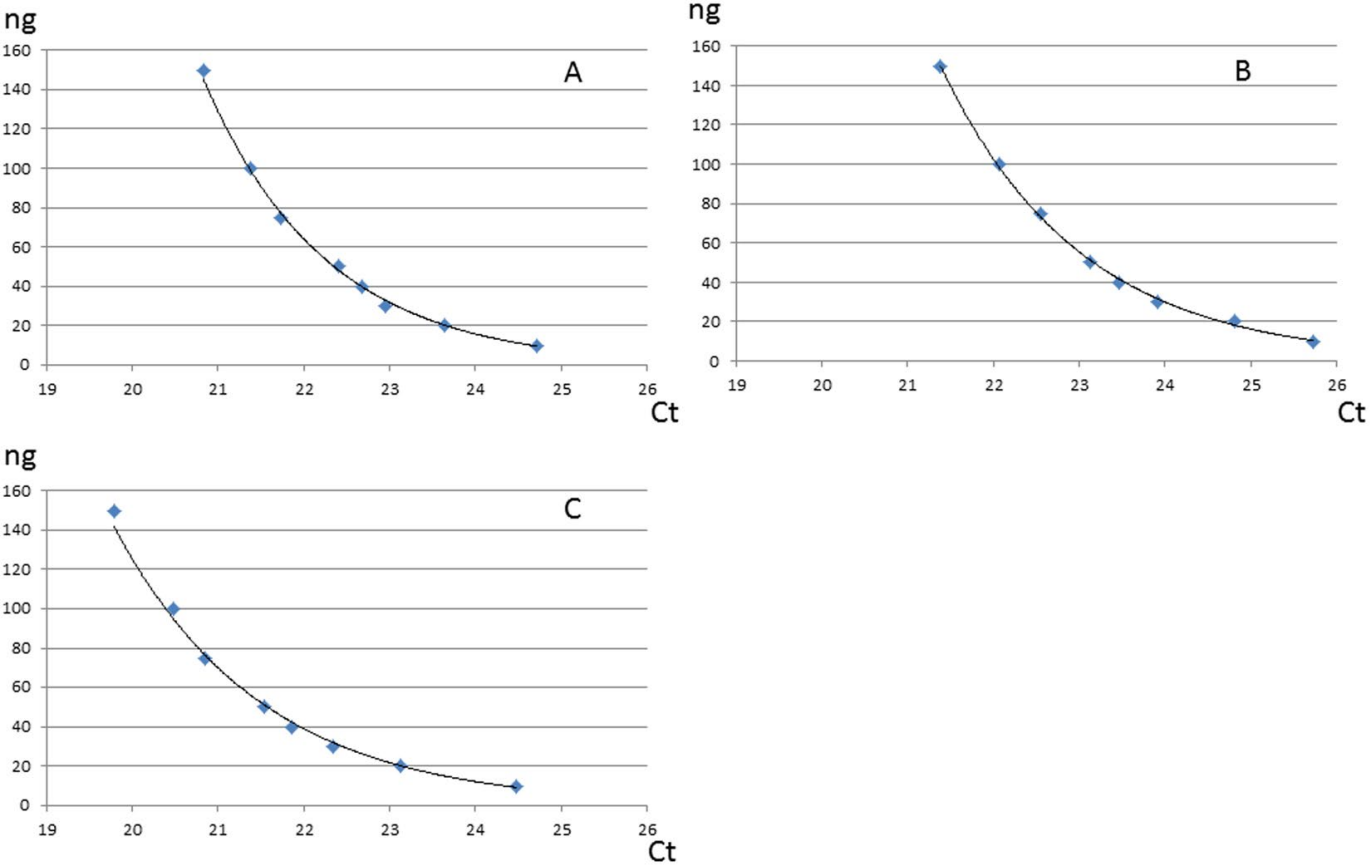
Sensitivity curves for oral cytobrush samples from patients with known oral HPV contamination for HR HPV (**A**), HPV16 (**B**) and HPV18 (**C**). Ten nanograms of DNA are sufficient to allow HPV DNA detection with the Cobas assay.

Detection of HPV was possible in cytobrush and FFPE samples with a minimal DNA input of 10 ng (Fig. 1) with Ct not higher than 35, except for HPV 16 FFPE sample (Ct [38.18; 44.9]).

No impact on the final result has been found for volumes from 14 to 16.9 µL for MMX and 8.1 to 12 µL for Mn/Mg for cytobrush and FFPE samples.

### Assays comparison

Among the 53 samples of patients with head and neck cancer, 9 did not have interpretable results with conventional PCR and 8 with Cobas assay. Thus 44 samples were considered for assay comparison. One sample had HPV 6/11 DNA which was detected with conventional PCR and not with Cobas assay. One sample had HPV 16 DNA detected with Cobas assay and not with conventional PCR. Calculated sensitivity and specificity of Cobas assay are 91.7% [61.5%; 99.8%] and 96.9% [83.8%; 99.9%] respectively.

Among the 26 samples of patients with anogenital neoplasia, all had interpretable results with both assays. One sample had HPV High Risk DNA which was detected with conventional PCR and not with Cobas assay. One sample had HPV 16 DNA detected with Cobas assay and HPV 16 and HPV HR DNA with conventional PCR. One sample had HPV 16 DNA detected with Cobas assay and not with conventional PCR and one sample had HPV 18 DNA detected with Cobas assay and not with conventional PCR. Calculated sensitivity and specificity of Cobas assay are 86.7% [59.5.5%; 98.3%] and 81.8% [48.2%; 97.7%] respectively.

Calculated sensitivity and specificity for samples of head and neck cancer and anogenital neoplasia (n = 70) were 88.9% [70.8%; 97.6%] and 93.0% [80.9%; 98.5%] respectively (Table 2).

**Table 2:** Calculated sensitivities and sensibilities.

	Sensitivity	Specificity
Head and neck cancer samples	91.7% [61.5%; 99.8%] (11/12)	96.9% [83.8%; 99.9%] (31/32)
Anogenital neoplasia samples	86.7% [59.5%; 98.3%] (13/15)	81.8% [48.2%; 97.7%] (9/11)
All samples	88.9% [70.8%; 97.6%] (24/27)	93.0% [80.9%; 98.5%] (40/43)

## Discussion

HPV infection is now clearly associated with an increasing incidence in oropharyngeal squamous cell carcinoma. The young adult population, non-smoker and non-drinker, is particularly exposed to these HPV-related cancers^11,12^. There are currently no clinical guidelines differentiating the treatment of HPV positive and HPV negative cancers^13,14^. However, research suggests that HPV expression is correlated with increased response rates to conventional treatments as radiotherapy, chemotherapy and radiochemotherapy^15–17^. Moreover, patients with HPV-associated head and neck squamous cell carcinoma appear to be the most appropriate subgroup to benefit from minimally invasive trans-oral surgery^13,18^. HPV status of these tumors will probably determine their management, thus HPV detection in head and neck cancer is highly important.

The Cobas real-time PCR assay validation data proved that this method is suitable for the detection of HPV DNA in oral cytobrush and FFPE samples with a very high repeatability and reproducibility. Moreover, the assay was really sensitive and able to detect HPV DNA with a low DNA input of 10 ng. Robustness of the assay was also very good because no impact on the final result has been found when sample, mix or coenzyme volumes were modified.

Only 12 HNC samples were positive, thus we chose to complete our study with 26 consecutive samples from patients with anogenital neoplasia. We assumed that most samples will be positive according to the HPV prevalence in this disease. We decided to not select only positive samples to also estimate the specificity of the Cobas assay in this cohort. Cobas Real-time PCR assay results were comparable to conventional PCR assay with a good sensitivity and specifiity for head and neck samples (91.7% [61.5%; 99.8%] and 96.9% [83.8%; 99.9%] respectively) and anogenital neoplasia FFPE samples (86.7% [59.5.5%; 98.3%] and 81.8% [48.2%; 97.7%] respectively). Some discrepancies were found in our study. One is related to the HPV types. HPV 6/11 DNA has been detected with conventional PCR and not with Cobas assay. This difference is explained by the design of the Cobas assay which is only based on the HR-HPV detection and HPV 6 and 11 are associated to low-risk. Whereas HPV 6 and 11 are considered as low risk HPV, they have been described as an etiological factor in the development non tumoral lesions, such as genital condylomas^1,19^ and laryngeal papillomatosis which may lead to carcinogenesis^20,21^. More recently, different studies showed that these two types are also involved in the development of anal dysplasia and cancers especially in men who have sex with men^22,23^. Two samples had HPV 16 DNA and one sample had HPV 18 DNA with Cobas assay and not with conventional PCR assay. We think that this result could be explained by a lower sensitivity of the conventional PCR assay, or could also be false positive; the Cobas HPV assay is based on TaqMan probes technology which has been reported to have no or few false positive results^24,25^. The last discrepant sample had only HPV 16 DNA with Cobas assay and HPV 16 and HPV HR DNA with conventional PCR. This discrepancy suggests that the sample could contain a double contamination with HPV 16 and another HPV with a genotype detected with the consensus primers of conventional PCR and not present in the consensus probes design of the Cobas assay.

Finally, in our study Cp were lower for FFPE samples for the detection of High Risk HPV and HPV 16 than oral cytobrush samples and higher for HPV18. These results can be explained by the DNA quality used in this study. Quality of DNA extracted from FFPE tissues depends on the duration of fixation and sample conservation whereas DNA of oral cytobrush samples have been extracted less than one week after collection^8^.

Our study has some limitations: First of all, the detection of HPV in our samples is limited by the assays we used. Cobas assay can only detect high risk HPV and conventional PCR can only detect high risk and 6/11 HPV, thus the term “absence of high risk HPV DNA and 6/11 low risk HPV DNA” would be more exact than “no HPV DNA”, especially for samples which are non-tumour or non-neoplasia. Moreover, HPVs are fast evolving viruses with different variants found in different populations. Cobas assay uses concensus probes for HR HPVs and since no sequencing has been done in our study, we can’t affirm that Cobas assay may be able to detect all variants subtypes.

Another technical limitation of the Cobas assay is the absence of copy number determination. It has been described that the copy number of the HPV infection will determine the duration and intensity of the therapies that will have minimal side effects but yet effective^26^. Moreover, the sensitivity has been evaluated using quantity of DNA but not with HPV copy number, thus our study only allows an estimation of the limit of detection of the assay. We assume that the DNA we used for this determination contained human and HPV DNA. Hence, it would make more sense if the internal controls with known copy numbers could be used to gauge the sensitivity on the basis of copy numbers rather than total DNA concentration.

This second assays comparison allowed showing that Cobas assay results were comparable to conventional PCR results. Another limitation is that 100% of our samples found with HPV DNA were HPV16 and HPV18, which is consistent with HPV prevalence in European countries. Samples with other high risks HPV would have been useful to demonstrate that Cobas assay was able to detect other genotypes. Moreover, Cobas assay does not allow the exact genotyping of non 16 or 18 HPV. Several described assays like SPF10-PCR DEIA LiPA25, Inno-LiPA, Linear Array or Onclarity allow the identification of more genotypes even on FFPE samples^27–29^. Finally, a capture NGS based assay has recently been described which allows the detection of 245 genotypes and the identification of viral insertion signature in human genome^30^.

In conclusion, we validated Cobas HPV test for the detection of HPV in FFPE samples and oral cytobrush samples. This assay is suitable for routine analysis and allows the detection of HPV DNA with a comparable sensitivity to conventional PCR.

## Methods

### Patients and samples

Fifty-three samples from 44 patients (median age 64.2 [29; 93]; sex ratio M/F 0.82) treated for a head and neck at Institut de Cancérologie de Lorraine, were prospectively collected for this study.

Among the 53 samples, 19 were oral cytobrush samples and 34 were FFPE tumor samples. Samples characteristics are detailed in Suppl Table 2-a.

Oral cytobrush samples were assessed using cervexbrush^®^ (Therapak corporation, Duarte, CA). Brush was introduced in oral cavity and brushed 5 times on internal side of the cheek, mobile tongue and anterior pillar of the soft palate or on the tumoral lesion. The brush-head was then conserved in Thinprep^®^ (Cytyc Corp, Marlborough, MA) at room temperature. Twenty-six consecutive FFPE samples from patients with anogenital neoplasia were retrospectively used for confirmation (Suppl Table 2-b).

Written informed consent was obtained from each patient involved in this study and the study has been validated by the scientific and ethic board of Institut de Cancérologie de Lorraine. All methods were performed in accordance with the relevant guidelines and regulations.

### DNA extraction

For FFPE samples, two 5 µm-thick serial sections were cut from each paraffin block and collected in Eppendorf^®^ vials. DNA was then extracted using Roche Cobas DNA Sample preparation Kit (Roche Diagnostics, Meylan, France) according to the manufacturer protocol.

Cytobrush samples collected in ThinPrep^®^ were centrifugated at 2000 rpm for 10 minutes at room temperature. The samples were then washed with PBS and centrifugated again at 2000 rpm for 10 minutes at room temperature. Supernate was eliminated and sediment was treated using 70 µL of proteinase K. DNA was finally isolated using Roche Cobas DNA Sample preparation Kit according to manufacturer protocol.

### DNA quality control

DNA quality was assessed using GAPDH housekeeping gene amplification (Forward 5′-TGG GGA AGG TGA AGG TCG GA-3′; Reverse 5′-GGG ATC TCG CTG GAA GA-3′) and gel migration as previously described^31^. DNA concentration was finally measured using NanoVue spectrophotometer (GE Healthcare, Buc, France).

### Real-time PCR procedure

**Figure 2:**
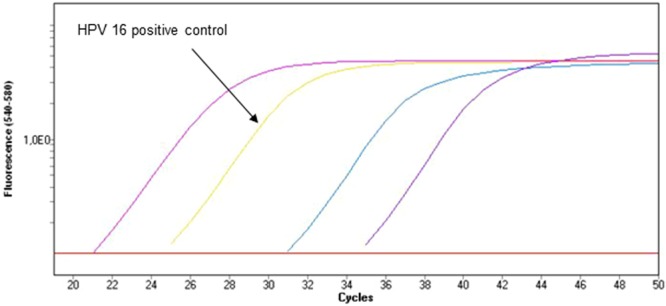
Amplification curves for a sample with HPV 16 DNA. Positive and amplification controls are also used for interpretation. An amplification is synonym of presence of HPV DNA.

Detection of HPV 16, 18 and consensus high risk HPV (31, 33, 35, 39, 45, 51, 52, 56, 58, 66 and 68) was assessed using Cobas HPV test (Roche Diagnostics, Meylan, France) based on TaqMan probes technology and Cobas z480 (Roche Diagnostics). The Cobas HPV test is a CE-IVD validated assay for the detection of HPV in cervical cytobrush samples but is not designed for the detection of oral cytobrush or FFPE samples^32,33^. A total of 50 µL of reactional mix constituted of 16.9 µL of HPV MMX, 8.1 µL of HPV Mn/Mg and 25 µL of samples was added in a 96 wells plate. β-globin was used as housekeeping gene. Internal controls (HPV 16, 18, high risk consensus and β-globin plasmids) were used on each plate. PCR consisted in 50 cycles (93 °C and 56 °C) followed by 30 seconds of cooldown to 40 °C and 10 seconds at 25 °C. Data were finally assessed using LightCycler 480 SW 1.5.0 software (Roche Diagnostics) (Fig. 2).

### Real-time PCR assay validation

Repeatability, reproducibility, sensitivity and robustness were assessed for the validation of Cobas real-time PCR assay for oral cytobrush and tumor samples.

Repeatability was measured for each HPV genotype (16, 18 and High risk) and for β-globin for oral cytobrush samples and tumor. For each sample (one FFPE and one oral cytobrush sample for HPV 16, HPV 18 and HPV HR) crossing point-PCR-cycle (Cp) was measured 10 times during the same run. The mean, the standard deviation and coefficients of variation (CV) were calculated for each genotype and sample type.

One oral cytobrush sample (HPV 16, HPV 18 and HPV HR) and one FFPE sample (HPV 16, HPV 18 and HPV HR) have been analyzed 15 times (5 times per run in 3 different runs on a total period of 3 weeks) and Cp for HPV and for β-globin were measured to estimate the reproducibility of the assay. The mean, the standard deviation and coefficients of variation (CV) were calculated for each genotype and sample type.

Limit of detection has been tested using control dilution from 0.4 ng/µL to 6 ng/µL for each HPV genotype and for each type of samples (FFPE and oral cytobrush), equivalent to a total DNA input of 10, 20, 30, 40, 50, 75, 100 and 150 ng. Samples from patients with HNC and a known oral HPV infection were used for this determination.

Finally, robustness was evaluated using variations of MMX reagent from 14 to 20 µL and Mg/Mn reagent from 7 to 12 µL.

### Conventional PCR

Specific primers for HPV 6/11, 16, 18, 33 and consensus (HPV 6, 11, 13, 15, 30, 31, 32, 33, 34, 35, 39, 40, 42, 45, 51, 52, 53, 56, 58, 61, 66 and 80) were used for the detection of HPV presence in samples. All primers are detailed in Suppl Table 3.

Migration was assessed using 1% agarose gel with 0.5X TBE and BlueJuice^TM^ gel loading buffer (ThermoFisher Scientific, Villebon-sur-Yvette, France) at 100 Volts for 2 hours. A total volume of 10 µL of each sample was finally used for HPV assessment and a DNA size marker (Roche Diagnostics) was present on each gel. Gels were finally analyzed using Gel DocEQ (BioRad, Marne-la-coquette, France). Conventional PCR with electrophoresis procedure are then mentioned as “Conventional PCR” in this manuscript.

### Statistical analysis and assays comparison

Repeatability and reproducibility were evaluated by the coefficient of variation (CV) calculated by dividing the standard deviation by the mean.

Conventional PCR assay was set as the gold standard for this study. The sensitivity for Cobas assay corresponded to the number of positive samples with Cobas assay divided by the number of positive samples with the conventional PCR assay. The specificity for Cobas assay corresponds to the number of negative samples with Cobas divided by the number of negative samples with the conventional PCR assay. The 95% confidence intervals for sensitivity and specificity were computed.

Statistical analyses were performed with SAS software version 9.2 (SAS Institute Inc., Cary, NC27513 USA). The assay comparison has first been assessed using only head and neck tumor samples (oral cytobrush and FFPE) and has been confirmed using 26 FFPE anogenital neoplasia samples.

## Electronic supplementary material

Supplementary tables
