# Supplementary material for: Evaluation and validation of HPV real-time PCR assay for the detection of HPV DNA in oral cytobrush and FFPE samples
Source: Sci Rep. 2018 Jul 27;8:11313. doi: 10.1038/s41598-018-29790-z (PMC6063863; doi:10.1038/s41598-018-29790-z)
Supplement: Supplementary file 1 — Supplementary tables [file 41598_2018_29790_MOESM1_ESM.docx]

**Evaluation and validation of HPV real-time PCR assay for the detection of HPV DNA in oral cytobrush and FFPE samples**

Alexandre Harlé^1,2,3,*^, Julie Guillet^1,2,4,*^, Jacques Thomas^3^, Xavier Sastre-Garau^3^, Marie Rouyer^3^, Carole Ramacci^3^, Pauline Gilson^1,2,3^, Cindy Dubois^3^, Gilles Dolivet^1,2,4^, Agnès Leroux^3^, Julia Salleron^5^, Jean-Louis Merlin^1,2,3^

^1^ Université de Lorraine, Nancy, France

^2^ CNRS, UMR 7039 CRAN, Nancy, France

^3^ Service de Biopathologie, Institut de Cancérologie de Lorraine, Vandœuvre-lès-Nancy, France

^4^ Unité de chirurgie cervico-faciale et odontologie, Institut de Cancérologie de Lorraine, Vandœuvre-lès-Nancy, France

^5^ Cellule data management et Biostatistique, Institut de Cancérologie de Lorraine, Vandœuvre-lès-Nancy, France

^*^ contributed equally to this paper

***Corresponding author:***

Dr. Alexandre Harlé

Service de Biopathologie, Institut de Cancérologie de Lorraine

6 Avenue de Bourgogne, CS 30519

54519 Vandoeuvre-lès-Nancy Cedex

France

[a.harle@nancy.unicancer.fr](mailto:a.harle@nancy.unicancer.fr)

Tel. +33 383 598 673 / Fax. +33 383 598 569

***Running title:*** HPV Real-time PCR detection

**Suppl Table 1:** High risk (HR) and Low risk (LR) oncogene HPV classification according to International Agency for Research on Cancer.

| Classification | Types |
| --- | --- |
| HPV-HR | 16, 18, 33, 34, 35, 39, 45, 51, 52, 56, 58, 59 |
| HPV-LR | 6, 11, 13, 40, 42, 43, 44, 54, 61, 70, 72, 80, 89 |

**Suppl Table 2-a:** Head and neck cancer samples characteristics

| # | **Sample type** | **Localization** | **Histology/cytology** | **Cobas result** | **Conventional PCR result** |
| --- | --- | --- | --- | --- | --- |
| 1 | FFPE | Lymph node | Metastatic SCC | HPV16 | HPV 16 |
| 2 | FFPE | Molar tooth | Ameloblastoma | - | - |
| 3 | FFPE | Lips | SCC | - | - |
| 4 | FFPE | Oropharynx (Tonsil pillar) | High grade intraepithelial neoplasia | HPV HR | HPV 16 |
| 5 | FFPE | Maxilla | SCC | - | - |
| 6 | FFPE | Tongue | SCC | - | - |
| 7 | FFPE | Tongue | SCC | - | - |
| 8 | FFPE | Oropharynx | SCC | HPV 16 | HPV 16 |
| 9 | FFPE | Posterior pharyngeal wall | SCC | - | invalid |
| 10 | FFPE | Oropharynx (base of Tongue) | SCC | - | - |
| 11 | FFPE | Nose | SCC | - | - |
| 12 | FFPE | Hypopharynx (Piriform sinus) | SCC | HPV 16 | HPV 16 |
| 13 | FFPE | Oropharynx | SCC | - | - |
| 14 | FFPE | Tonsil | SCC | HPV 16 | HPV 16 |
| 15 | FFPE | Tonsil | SCC | HPV 16 | HPV 16 |
| 16 | FFPE | Floor of the mouth | SCC | - | - |
| 17 | FFPE | Retro-molar area | SCC | - | - |
| 18 | FFPE | Oral tongue | No malignancy | INVALID | INVALID |
| 19 | FFPE | Floor of the mouth | SCC | - | - |
| 20 | FFPE | Piriform sinus | SCC | INVALID | INVALID |
| 21 | FFPE | Tonsil | SCC | HPV 16 | HPV 16 |
| 22 | FFPE | Floor of the mouth | SCC | - | - |
| 23 | FFPE | Nasopharynx | No malignancy | - | - |
| 24 | FFPE | Oropharynx | SCC | HPV 16 | HPV 16 |
| 25 | FFPE | Upper jaw | No malignancy | - | - |
| 26 | FFPE | Larynx | Papilloma | - | HPV 6/11 |
| 27 | FFPE | Maxillaire | Warty carcinoma | - | - |
| 28 | FFPE | Tonsil | SCC | HPV 16 | HPV 16 |
| 29 | FFPE | Nasopharynx | SCC | - | - |
| 30 | FFPE | Nasopharynx | Undifferentiated carcinoma | INVALID | INVALID |
| 31 | FFPE | Vocal cord | SCC | - | - |
| 32 | FFPE | Hypopharynx | SCC | - | - |
| 33 | FFPE | Tonsil | No malignancy | - | - |
| 34 | FFPE | Larynx | SCC | - | - |
| 35 | oral cytobrush sample | Oropharynx | No malignancy | - | - |
| 36 | oral cytobrush sample | Soft palate | No malignancy | - | - |
| 37 | oral cytobrush sample | Oral cavity (cheeks) | No malignancy | - | - |
| 38 | oral cytobrush sample | Oral cavity (cheeks) | No malignancy | INVALID | INVALID |
| 39 | oral cytobrush sample | Oropharynx | No malignancy | INVALID | INVALID |
| 40 | oral cytobrush sample | Oral cavity (cheeks) | No malignancy | - | - |
| 41 | oral cytobrush sample | Oropharynx | No malignancy | HPV 16 | HPV 16 |
| 42 | oral cytobrush sample | Oral cavity (cheeks) | Mild cytonuclear abnormalities | - | - |
| 43 | oral cytobrush sample | Oral cavity (cheeks) | Squamous cells without malignancy. | - | - |
| 44 | oral cytobrush sample | Oral cavity (cheeks) | No malignancy | - | - |
| 45 | oral cytobrush sample | Oral cavity (cheeks) | No malignancy | - | - |
| 46 | oral cytobrush sample | Oral cavity (cheeks) | No malignancy | - | - |
| 47 | oral cytobrush sample | Oropharynx | No malignancy | - | - |
| 48 | oral cytobrush sample | Oral cavity (cheeks) | Low grade Intra epithelial neoplasia | INVALID | INVALID |
| 49 | oral cytobrush sample | Oral cavity (cheeks) | No malignancy | INVALID | INVALID |
| 50 | oral cytobrush sample | Oral cavity (cheeks) | Mild cytonuclear abnormalities | HPV 16 | HPV 16 |
| 51 | oral cytobrush sample | Oral cavity (cheeks) | Squamous cells with parakeratosis | HPV 16 | - |
| 52 | oral cytobrush sample | Oral cavity (cheeks) | No malignancy | INVALID | INVALID |
| 53 | oral cytobrush sample | Tonsil pillar | No malignancy | - | - |

**Suppl Table 2-b:** Anogenital neoplasia samples characteristics

| # | **Sample type** | **Localization** | **Histology** | **Cobas result** | **Conventional PCR result** |
| --- | --- | --- | --- | --- | --- |
| 1 | FFPE | Uterine cervix | CIN3 | - | HPV HR |
| 2 | FFPE | Vulva | VIN1 | HPV18 | HPV18 |
| 3 | FFPE | Uterine cervix | AIS | HPV 16 HPV 18 | HPV16 HPV 18 |
| 4 | FFPE | Vulva | VIN2 | - | - |
| 5 | FFPE | Vulva | VIN2 | - | - |
| 6 | FFPE | Vagina | SCC | HPV16 | HPV16 |
| 7 | FFPE | Uterine cervix | ADC | HPV16 | HPV16 |
| 8 | FFPE | Endometrium | Serous ADC | - | HPV HR |
| 9 | FFPE | Anal margin | SCC | HPV16 | HPV16 |
| 10 | FFPE | Bladder | SCC | - | - |
| 11 | FFPE | Uterine cervix | CIN1 | HPV HR | HPV HR |
| 12 | FFPE | Uterine cervix | CIN1 | HPV HR | HPV HR |
| 13 | FFPE | Uterine cervix | SCC | HPV16 | HPV HR HPV 16 |
| 14 | FFPE | Anal margin | AIN3 | HPV16 | HPV16 |
| 15 | FFPE | Uterine cervix | ADC | HPV18 | HPV18 |
| 16 | FFPE | Uterine cervix | ADC | HPV18 | HPV18 |
| 17 | FFPE | Vulva | SCC | - | - |
| 18 | FFPE | Uterine cervix | SCC | - | - |
| 19 | FFPE | Endometrium | polymorphous ADC | HPV18 | - |
| 20 | FFPE | Uterine cervix | ADC | HPV16 | - |
| 21 | FFPE | Uterine cervix | CIN3 | HPV16 | HPV16 |
| 22 | FFPE | Uterine cervix | ADC | - | - |
| 23 | FFPE | Vulva | SCC | - | - |
| 24 | FFPE | Anal Canal | SCC | - | - |
| 25 | FFPE | Anal margin | AIN3 | HPV16 | HPV16 |
| 26 | FFPE | Endometrium | ADC | - | - |

**Suppl Table 3:** Conventional PCR primers

| **Genotype** | **Forward primer** | **Reverse primer** |
| --- | --- | --- |
| HPV consensus | 5’ - TTT GTT ACT GTG GTA GAT ACT AC - 3’ | 5’ - GAA AAA TAA ACT GTA AAT CAT ATT C - 3’ |
| HPV 6/11 | 5’ - TTG CAC TAT AGG CGT AGC TG - 3’ | 5’ - ATG TTA TGG CAG CAC AGT TA - 3’ |
| HPV 16 | 5’ - GTG GAC CGG TCG ATG TAT GT - 3’ | 5’ - CAT GCA ATG TAG GTG TAT CT - 3’ |
| HPV 18 | 5’ - GCA GCA CAG AAA ACA GTC CA - 3’ | 5’ - CGC CAT TTG TAG TTA CCT GA - 3’ |
| HPV 33 | 5’ - AGT CAA AAT GGC GAC ACA AA - 3’ | 5’ - ACT AAT TTC CTG CAA CGT AA - 3’ |
